# Cyclic AMP signaling restricts activation and promotes maturation and antioxidant defenses in astrocytes

**DOI:** 10.1186/s12864-016-2623-4

**Published:** 2016-04-23

**Authors:** Sonia Paco, Manuela Hummel, Virginia Plá, Lauro Sumoy, Fernando Aguado

**Affiliations:** Department of Cell Biology, University of Barcelona, Av. Diagonal 645, Barcelona, E-08028 Spain; Centre for Genomic Regulation, Barcelona, E-08003 Spain; Institute of Neurosciences, University of Barcelona, Barcelona, E-08035 Spain; Institute for Predictive and Personalized Medicine of Cancer, Badalona, E-08916 Spain; Germans Trias i Pujol Health Sciences Research Institute, E-08916 Badalona, Spain; Present address: Department of Pediatric Hematology and Oncology, Hospital Sant Joan de Déu Barcelona, Esplugues de Llobregat, E-08950 Spain; Present address: Division of Biostatistics, German Cancer Research Center, Im Neuenheimer Feld 581, D-69120 Heidelberg, Germany

**Keywords:** Antioxidant defense, Astrocytes, cAMP, Differentiation, Reactive glia, Transcriptomic, Brain, NR2C

## Abstract

**Background:**

cAMP signaling produces dramatic changes in astrocyte morphology and physiology. However, its involvement in phenotype acquisition and the transcriptionally mediated mechanisms of action are largely unknown.

**Results:**

Here we analyzed the global transcriptome of cultured astroglial cells incubated with activators of cAMP pathways. A bulk of astroglial transcripts, 6221 annotated genes, were differentially regulated by cAMP signaling. cAMP analogs strongly upregulated genes involved in typical functions of mature astrocytes, such as homeostatic control, metabolic and structural support to neurons, antioxidant defense and communication, whereas they downregulated a considerable number of proliferating and immaturity-related transcripts. Moreover, numerous genes typically activated in reactive cells, such as scar components and immunological mediators, were repressed by cAMP. GSEA analysis contrasting gene expression profiles with transcriptome signatures of acutely isolated astrocytes and *in situ* evaluation of protein levels in these cells showed that cAMP signaling conferred mature and in vivo–like transcriptional features to cultured astrocytes.

**Conclusions:**

These results indicate that cAMP signaling is a key pathway promoting astrocyte maturation and restricting their developmental and activation features. Therefore, a positive modulation of cAMP signaling may promote the normal state of differentiated astrocytes and favor the protection and function of neuronal networks.

**Electronic supplementary material:**

The online version of this article (doi:10.1186/s12864-016-2623-4) contains supplementary material, which is available to authorized users.

## Background

Mature astrocytes ensheath synapses and fine blood vessels, within the neuro-glio-vascular units, to shape the functional micro-architecture of the central nervous system (CNS). Through an array of transporters, ion channels and released and adhesion molecules, astrocytes play key roles in the regulation of extracellular fluid homeostasis, integrity of the blood–brain barrier and assurance of metabolic demand and antioxidant defense of neurons. Astrocytes also make a crucial contribution to communication, operating within glial networks through gap junctions and hemichannels and bidirectionally with neurons and endothelial cells via diffusible and surface molecules [1–4]. Characteristically, in response to CNS insults, such as trauma, epilepsy or degenerative diseases, astrocytes become reactive and play a critical role in neuroinflammation and scar formation [4].

In contrast to neurons, little is known about the intracellular signaling of astrocytes, and the pathways controlling their differentiation and activation have not been elucidated. By means of STAT transcription factors, cytokines of the interleukin-6 family are key cues in the specification and differentiation of astroglial cells. Moreover, Smad transcription factors activated by bone morphogenetic proteins also promote astrogliogenesis, through their interaction with STAT3 [5]. Strikingly, analysis of glial activation in pro-inflammatory cytokine-administered and genetically-modified mice has shown that JAK-STAT3 signaling is also a key pathway through which astrocytes become reactive [6]. In addition to activating kinase-dependent receptors, G-protein coupled receptors can trigger astroglial genesis and maturation via cAMP [7–11]. Changes in cell morphology and protein expression induced by cAMP have long been considered a feature of astrocyte activation [12–16]. However, the precise significance of the cAMP pathway in the maturation and activation of these cells remains elusive. Here we studied the net impact of the cAMP signaling pathway on astrocytes by analyzing the global transcriptome of cultured cells incubated with permeable cAMP analogs. Moreover, we perform Gene Set Enrichment Analysis (GSEA) of cAMP-regulated genes and contrasted our findings with recent microarray reports and the in vivo expression patterns of astrocytes. Our findings show that the overall impact of the cAMP signaling pathway in astrocytes is to promote maturation and restrict developmental and activation features.

## Results and discussion

### Genome-wide transcriptional regulation in astrocytes by cAMP signaling

**Fig. 1:**
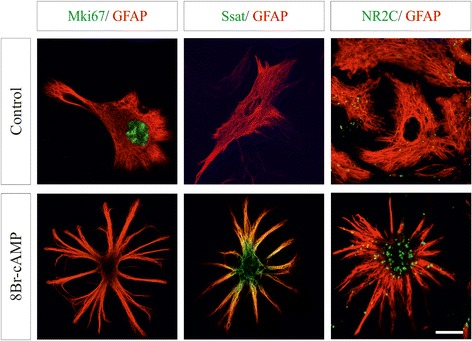
Regulation of microarray expression in identified single astrocytes in culture. Double immunofluorescence confocal images of the astrocyte marker GFAP (red) and three proteins differentially regulated by cAMP (green) in control and treated cells. Mki67 and Ssat were analyzed in permeabilized astrocytes and NR2C in non-permeabilized cells. Nuclear Mki67 immunfluorescence was abundant in control astrocytes, but almost absent in cAMP-treated cells. Diffuse cytosolic Ssat and extracellular clusters of NR2C were intensely labeled in treated cells. Yellow signal in the Ssat labeling indicates colocalization with GFAP cytoskeleton. Note the star-shaped morphology acquired by astrocytes exposed to permeable cAMP agonists. Scale bar: 10 μm

To activate cAMP signaling we augmented intracellular levels by exposing cultured astrocytes to the permeable cAMP analog 8Br-cAMP. Before and after treatment cell cultures were essentially formed by astrocytes (>95 % GFAP^+^ cells). Sustained elevated intracellular cAMP levels altered the number and morphology of astrocytes. Quantitative analysis showed almost 25 % less GFAP^+^ astrocytes after treatment compared with controls (54.21 ± 0.78 cell/mm^2^ and 72.08 ± 1.24 cell/mm^2^, respectively, *p* = 0.006). In agreement with previous studies [7, 17], control flat polygonal-shaped astroglial cells were converted into process-bearing stellate astrocytes (Fig. 1). Microarray analysis showed that 6221 of 16,594 annotated genes with assigned gene symbol were differentially expressed by a factor of 1.2 or greater (and FDR q-value <0.05) in 8Br-cAMP-treated astrocytes. The full list of differentially expressed genes is shown in Additional file 1: Table S1. Approximately, one-third of the regulated elements on the array corresponded to ESTs and genes of unknown function. Of the 6221 genes with significant regulation 42.1 % were upregulated by cAMP while the rest were downregulated.

**Fig. 2:**
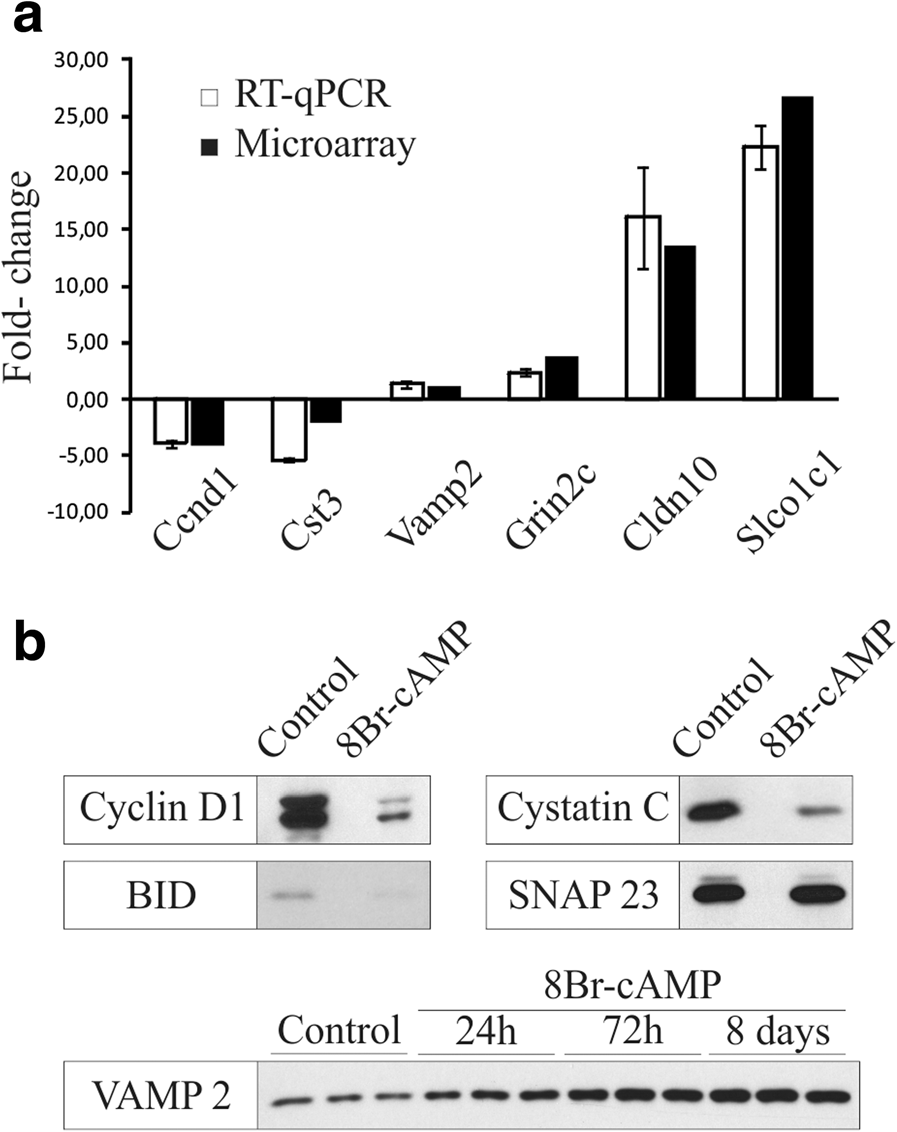
Validation of microarray expression data for selected differentially expressed genes in cultured astrocytes at the mRNA and protein levels. **a** Comparison of cDNA microarray and qRT-PCR data. **b** Western blotting analysis of cAMP-regulated genes in control and treated astrocytes

We validated the present array results by analyzing astrocyte expression of the mRNA and protein of 10 representative genes after cAMP augmentation (Figs. 1 and 2). qPCR analysis was performed on the cell cycle regulator cyclin D1 (*Ccnd1*), the peptidase inhibitor cystatin C (*Cst3*), the calcium-dependent exocytotic SNARE *Vamp2*, the NMDA receptor subunit NR2C (*Grin2c*), the adhesion component claudin 10 (*Cldn10*) and the thyroid hormone transporter *Slco1c1*. A correlation was found between RT-qPCR and array results for each gene (Fig. 2a). cAMP-induced changes of cyclin D1, cystatin C, Vamp2 and the apoptotic regulator Bid, as well as their time dependence, were further corroborate at protein level by immunoblotting (Fig. 2b). No changes on the array were found for non-regulated genes, as shown for the ubiquitous exocytotic protein Snap23 (Fig. 2b). Immunofluorescence for the proliferation marker Mki67, the polyamine enzyme Ssat (*Sat1*) and the NR2C receptor demonstrated that cAMP-dependent gene regulation occurred in GFAP-identified astrocytes (Fig. 1). Furthermore, previous studies showing that cAMP regulates the expression of intermediate filaments (GFAP and Nes), glutamate transporters (Slc1a2 and Slc1a3), deiodinases (Dio2), and secretogranins (Scg2 and Scg3) in astrocytes [8, 9, 18, 19] and Additional file 1: Table S1.

### cAMP-induced changes in functional expression profiles of astrocytes

**Fig. 3:**
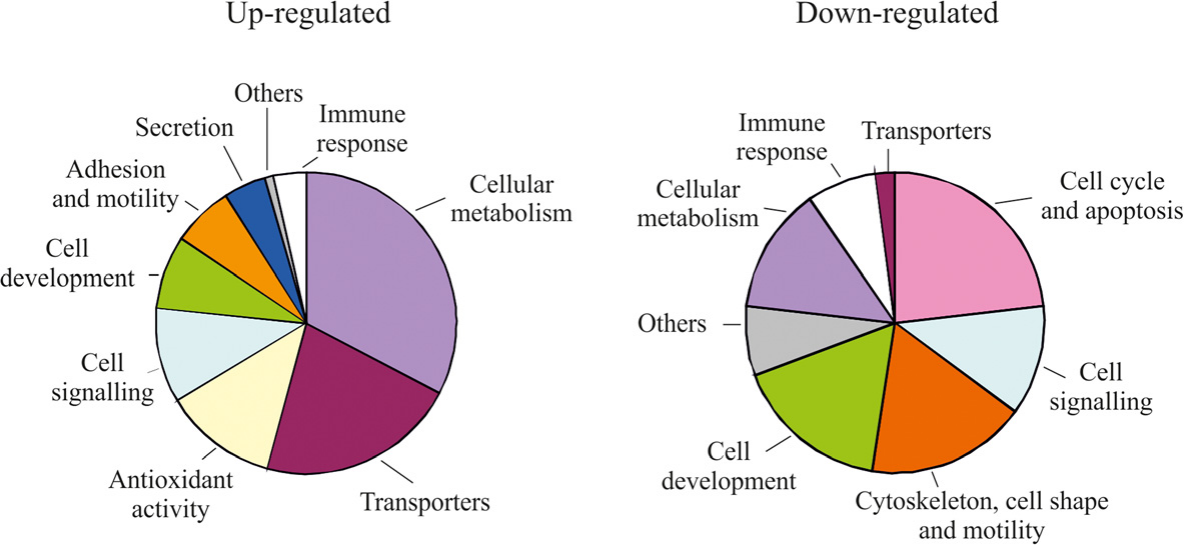
Global GO classification of gene expression profile of cAMP-treated astrocytes. Pie charts depicting the relative proportions of significant up- and downregulated genes in general categories containing distinctive GO terms. The GO analysis was performed using GSEA (http://software.broadinstitute.org/gsea/index.jsp). Detailed GO terms are shown in Additional file 2: Figure S1

The functional roles of up- and downregulated cAMP-responsive genes were categorized by Gene Ontology enrichment analysis using GSEA. GO categories with GSEA q-value <2 % were considered relevant. We calculated the relative proportion of genes in the core enrichment of each GO category and grouped them in general categories (Fig. 3). All the GO categories enriched with differentially regulated genes are shown in the bar plot of Additional file 2: Figure S1.

More than half (55.2 %) the upregulated genes in enriched GO categories corresponded to cell metabolism and transporters. Thus, genes involved in the uptake and degradation of neurotransmitters such as glutamate, glycine and catecholamines were overexpressed after treatment (*Slc1a2*, *Glul*, *Gldc* and *Maob*). Ion channels, pumps and transporters with key homeostatic and metabolic functions were also preferentially upregulated by cAMP. For instance, genes participating in the membrane transport of K^+^ (*Kcnn2*), Cl^−^ (*Clcn2*), Ca^2+^ (*Atp2a2* and *Atp2b2*), water (*Aqp4*), glucose (*Slc2a1*), thyroid hormones (*Slco1c1* and *Slc16a2*), and oligopeptides (*Slc15a2*) were activated by cAMP signaling. Moreover, an increase of cAMP levels induced expression of genes coding for a wide variety of astrocytic metabolic enzymes, such as most aldehyde dehydrogenases, tRNA ribonucleases and GPI transamidases. Interestingly, the third functional category of genes upregulated by cAMP was associated with antioxidant activities (Table 1). Thus, genes encoding for antioxidant enzymes and molecules, such as catalase, superoxide dismutases, metallothioneins, heme oxygenases and NADPH-quinone oxidoreductases were found to be through microarray analysis. Enhanced cAMP also upregulated numerous genes related to the glutathione system and transport of antioxidant vitamins. These results support previous studies showing an antioxidant involvement of the cAMP pathway in the CNS and suggest that cAMP signaling promotes glial differentiation and antioxidant protection [1, 20].

**Table 1:** Antioxidant-associated genes upregulated by cAMP in astrocytes (fold-change)

Catalase	Cat (2.2)
Superoxidase dismutases	Sod2 (1.4), Sod3 (5.3)
Peroxiredoxins and sulfiredoxins	Prdx1 (1.8), Prdx2 (1.5), Prdx5 (1.5), Srxn1 (4.1)
Cystine/glutamate transporter	Slc7a11 (9.6)
Glutamate-cysteine ligases	Gclc (5.3), Gclm (3.1)
Gluthatione synthetase and transferases	Gss (1.5), Gsta1 (15.7), Gsta2 (6.9), Gsta3 (6.6), Gsta4 (2.3), Gstm5 (1.6), Mgst1 (4.4), Mgst2 (7.7)
Glutathione peroxidases and reductases	Gpx4 (1.6), Gsr (1.5)
NADPH-quinone oxidoreductase and heme oxygenase	Nqo1 (4.4)
Thioredoxins and thioredoxin reductases	Txn2 (1.6), Txnrd1 (3.3)
Metallothioneins and selenoproteins	Mt1 (1.6), Mt2 (1.5), Sepp1 (1.3)
Ascorbate and glucose/dehydroascorbate transporters	Slc23a2 (4.5), Slc2a1 (3.9), Slc2a4 (1.4), Slc2a8 (1.7), Slc2a13 (2.2)
α-tocopherol transfer protein	Ttpa (2.2)

GO categories enriched with downregulated genes were associated mainly with proliferating and immaturity-related features of astrocytes (Table 2). Thus, 71.5 % of the genes were involved in the general categories cell cycle and apoptosis, cell signaling, cytoskeleton, cell shape and motility, and cell development. Expression of genes typically involved in mitotic processes was dramatically decreased, such as those encoding for the DNA polymerases and the proliferation antigen Mki67. Transcripts of proliferation and cell death regulators, such as mitogens and morphogens (IGF-1, PDGF and BMP4), cyclins, caspases and proapoptotic Bcl2 family members (e.i., Casp8, Bid and Bax) were reduced after exposure to cAMP. Moreover, numerous genes involved in cytoskeletal organization, such as Rho GTPases, myosins, actins, tropomyosins, kinesins and septins, were notably repressed. Among downregulated genes, those GO categories associated with reactive astrocytes such as components of the extracellular matrix and mediators of the immune response were predominantly enriched. Thus, mRNA expression of tenascin C, nidogens and many collagens and chondroitin and heparan sulfate proteoglycans was reduced. Interestingly, the enzymes responsible for the major postranscriptional modifications of proteoglycans and collagens were widely downregulated (sulfotransferases and lysyl oxidases). Finally, prolonged cAMP signaling stimulation in astrocytes induced a downregulation of numerous mediators of the immune response, such as cytokines, chemokines and components of the major histocompatibility complex and phagocytototic and adhesion processes (e.g., *Tnf*, *Ccl2*, *Tgfb1*, *Cxcl10*, *Csf1r*, *H2-D1*, *B2m*, *Gulp1* and *Icam1*). We conclude that cAMP contributes to the restriction of developmental and activation features of astrocytes.

**Table 2:** Selected categories and genes down-regulated by cAMP elevation in astrocytes

General category						
GO category	NES	FDR q-value	Gene	ID	Protein name	FC
Cell cycle and apoptosis						
Mitosis	−1,71	0.018	Ccna2	12428	cyclin A2	- 4,2
DNA directed DNA polymerase activity	−1,76	0.012	Pold1	18971	polymerase (DNA directed), delta 1, catalytic subunit	−2,3
Cytokinesis	−1,69	0.02	Myh10	77579	myosin, heavy polypeptide 10, non-muscle	−3,7
Cell signalling						
Ras protein signal transduction	−1,77	0.012	Igf1	16000	insulin-like growth factor 1	−6.1
Neuropeptide hormone activity	−1,29	0.183	Npy	109648	neuropeptide Y	- 3,5
Cytoskeleton, cell shape and motility						
Collagen	−1,75	0.013	Col5a2	12832	collagen type V a2	- 6,7
Structural constituent of cytoskeleton	−1,67	0.023	Tpm1	22003	tropomyosin 1, alpha	−3.1
Cell development						
Cell development	−1,85	0.008	Igfbp3	16009	insulin-like growth factor binding protein 3	- 4,1
Cellular metabolism						
Proteoglycan metabolic process	−1,76	0.012	Chst7	60322	chondroitin 6-sulfotransferase 7	- 2,9
Immune response						
Inflammatory response	−1,82	0.01	Ccl2	20296	chemokine (C-C motif) ligand 2	- 2,1
			Cxcl10	15945	chemokine (C-X-C motif) ligand 10	−3,7
Others						
Lipid raft	−2,01	0.0088	Cav1	12389	caveolin 1	- 4,0

### Elevated cAMP restricts the activation of astrocytes and promotes their differentiation

**Fig. 4:**
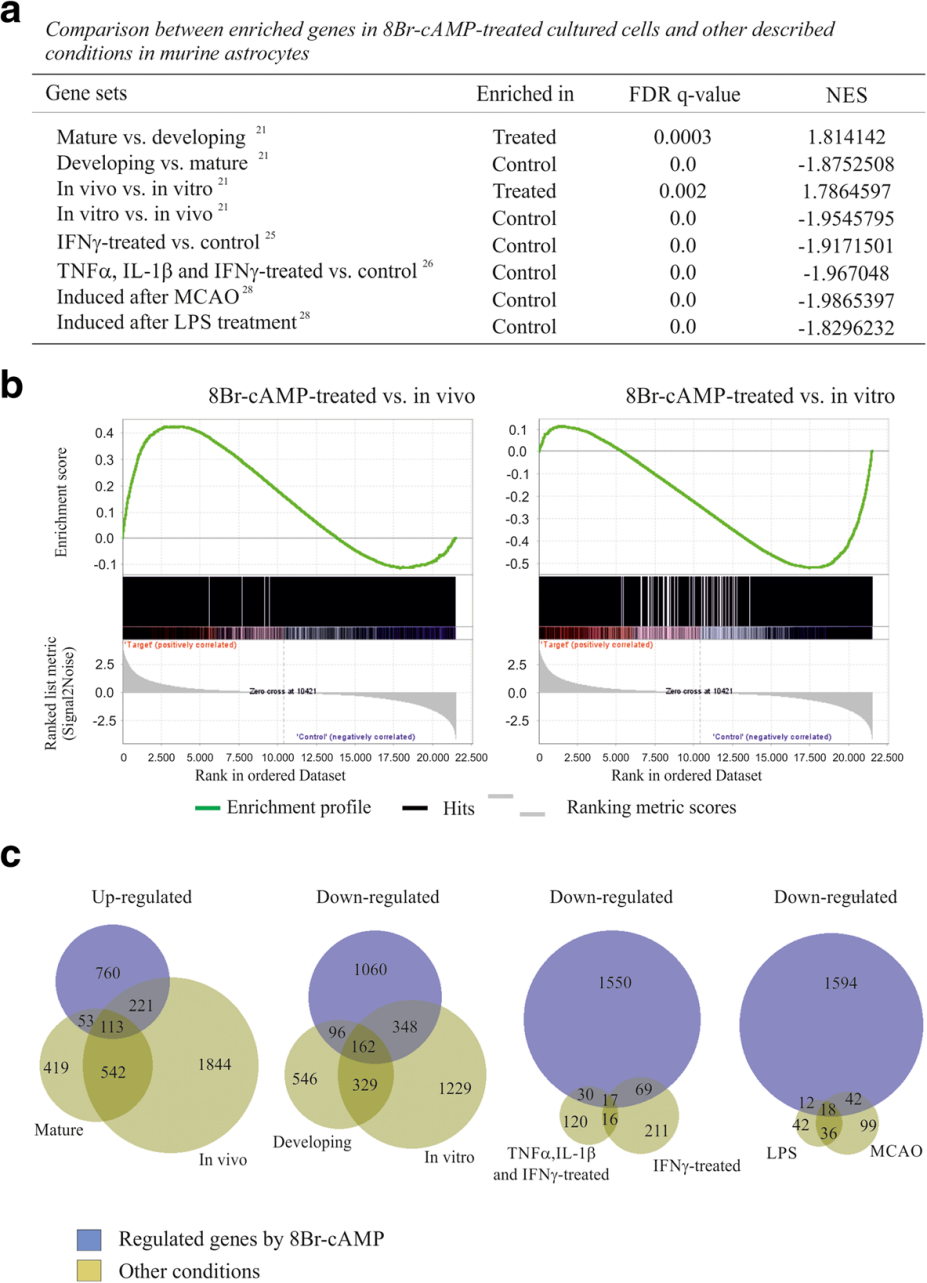
GSEA analysis comparing the gene profile of cAMP-treated astrocytes with the transcriptome signatures of astroglial cells in different states. **a** The upregulated gene set identified in cAMP-treated astrocytes is significantly enriched in mature and in vivo astroglial cells, while the cAMP-induced downregulated gene set is significantly enriched in developing, in vitro, proinflammatory cytokine-treated astrocytes and after MCAO and LPS treatment. FDR q-value, False discovery rate; NES, Normalized Enrichment Score. **b** Representative GSEA analysis showing the plots of cAMP-regulated gene profile with in vivo (*n* = 2821 genes) and in vitro (*n* = 2105 genes) gene sets obtained from Cahoy et al. 2008. Signal-to-Noise ratio statistic was used to rank the genes on the basis of their correlation with either the upregulation (red) or downregulation (blue). On each panel, the vertical black lines indicate the position of each gene of the set in the ordered, non-redundant data set. The green curve corresponds to the enrichment profile curve, which is the running sum of the weighted enrichment score obtained with GSEA software. **c** Proportional Venn diagrams representing the overlap of astroglial genes differentially expressed by cAMP compared to the gene sets in different astrocyte states (fold change cut-off 1.5 in each case except for LPS and MCAO that fold-change cut off was 4)

To substantiate the net phenotype induction of cAMP signaling on astroglial cells, we applied GSEA analysis to contrast the present gene profiles with previous microarray studies describing the transcriptome signatures of in vivo astrocytes acutely isolated in a range of states. First, we compared our genome profiling results with gene sets of developing and mature brain astrocytes reported by Cahoy et al. [21]. We found that cAMP-induced upregulated genes were significantly enriched for the mature profile of astrocytes (Fig. 4). The 166 common transcripts included metabolic (*Agxt2l1*), antioxidant (*Sod3*) and signaling (*Gjb2*). Conversely, 258 genes downregulated by cAMP, such as *Pold1*, *Ccna2* and *Npy*, were enriched for the developmental gene set of astrocytes (Fig. 4). The full lists are shown in Additional file 3: Table S2 and S3. We conclude that cAMP signaling causes the global transcriptome of astrocytes to become more differentiated. Previous electrophysiological and biochemical studies demonstrating that cAMP elevation induces the acquisition of mature astrocyte signatures, such as proliferation arrest, membrane conductances, neurotransmitter uptake and cytoskeletal construction further substantiate a relevant contribution of this pathway to astrocyte maturation [7–10]. In addition to supporting roles to neurons, astrocytes are dynamic regulatory elements implicated in the physiology of neural circuits [1–3]. The observed regulation of connexins and pannexins provides evidence for the crucial contribution of intracellular cAMP levels to glial crosstalk. Interestingly, cAMP augmentation also influences astroglial components of the so-called “tripartite synapse”. Here we show that the NMDA receptor subunit, NR2C, and the SNARE proposed to mediate astrocyte-to-neuron communication, VAMP-2 [22], were found to be under control of cAMP concentrations. These results together previous observations on regulated exocytosis implicate cAMP as a second messenger involved in neural communication [2, 17, 23].

Next, we compare our profiles of cAMP-regulated genes with genes overexpressed in activated astrocytes. First, we compared cAMP-regulated gene sets with previous studies performed on cultured astrocytes stimulated with pro-inflammatory cytokines [24, 25]. It has been established that in vitro administration of TNF-α, IL-1β and IFN-γ recapitulate many aspects of reactive astrogliosis, mainly those related to immunity processes [26]. Genes downregulated by cAMP were significantly enriched in upregulated gene sets of astrocytes treated with IFN-γ alone (*n* = 313 genes) or with a mix of TNF-α, IL-1β and IFN-γ (*n* = 183 genes) (Fig. 4a, c). Remarkably, more than a quarter of the genes upregulated by each inflammatory treatment were downregulated by cAMP. Gene sets downregulated by cAMP and upregulated by IFN-γ alone and by the cytokine mix shared 17 genes in common, 11 related to immune response (i.e. *Ccl2*, *Icam1*) and 4 to proteasomal and lysosomal components (i.e., *Ctsc*). The full gene list is in Additional file 3: Table S4. Consistently, similar opposite gene regulation was found comparing changes induced by cAMP with those produced by the inflammatory mediators TGF-β1, lipopolyssacaride (LPS) and IFN-γ on cultured astrocytes reported by Hamby et al. [27].

We further evaluated the activation state of astrocytes exposed to 8Br-cAMP comparing our results whit previous genomic analyses performed on reactive astrocytes acutely isolated from ischemic and LPS-exposed mouse brains [28]. As occurs in models of astrogliosis in vitro, genes repressed by cAMP were significantly enriched in activated gene sets of in vivo reactive astrocytes (Fig. 4a, c). Among the genes showing an opposite regulation in 8Br-cAMP-treated cells and brain isolated reactive astrocytes were those involved in cell cycle (*Ccnb1*, *Ccnd1*, *Gas1*, *Ki67*), immune response (*B2m*, *Ccl2*, *H2-K1*), extracellular matrix (*Anxa1*, *Anxa2*, *Col6a1*) and cell signaling and metabolism (*Cav1*, *Ch25h*, *Galntl2*, *Igfbp3*, *Klf6*, *Tnfrsf12a*). Stroke and neuroinflammation induce overlapping but distinct sets of activated genes in reactive astrocytes [28], thus around a third and quarter of the genes upregulated in vivo by ischemia and LPS, respectively, were significantly enriched for the profile of repressed by cAMP signaling (Fig. 4c, Additional file 3: Tables S5 and S6). Moreover, 7 out of the 18 genes downregulated by cAMP and activated by the two in vivo injury models were associated to immune response (Cd14, Cxcl10, Gbp3, Icam1, Iigp1, Oasl2 and Spp1). Taken together, these data indicate that cAMP signaling restrict reactive astrogliosis.

Our findings demonstrate a wide cAMP-induced downregulation of genes related to the main hallmarks of astroglial activation, such as hyperplasia, cytoskeletal rearrangement, triggering of immunological reactions and production of scar components. Many genes associated with proliferation and cytoskeletal components upregulated during activation were downregulated by cAMP (as shown for *Mki67* and *Nes*). This is expected because developing and reactive astrocytes share some cellular features. Moreover, cAMP elevation also represses a number of genes encoding for immunological mediators and scar components which are overexpressed in reactive astroglia (cytokines, chemokines, collagens, sulfated proteoglycans and adhesion molecules), including the new marker for reactive astrogliosis, podoplanin [29]. Although some transcripts implicated in glial activation were upregulated in cultured cells treated with 8Br-cAMP (i.e. *C3* and *Il-6*), our results suggest that cAMP signaling essentially represses astroglial activation. This conclusion may diverge from the assumption by some authors that cAMP analogs in cultured astrocytes promote activation [12–16]. However, this consideration was taken on the basis of changes in shape stellation and GFAP expression, which are also typical attributes of maturing astrocytes [8, 9, 30]. Noteworthy, our results showing an opposite transcriptome regulation by cAMP and glial-activating cytokines are consistent with previous studies demonstrating that elevated cAMP in astrocytes antagonizes the cytokine-induced expression of adhesion molecules involved in neuroinflammation [31]. A repressive role for cAMP in glial activation is also consistent with the observations that cAMP pathways are involved in the reduction and promotion of the glial activation triggered by anti-inflammatory and pro-inflamatory extracellular agents, respectively [32–34]. Moreover, the observation of cAMP-induced upregulation of repressors of the glial inflammatory response, such as *Socs1* (2.3 fold) and *Nr4a2* (10.5 fold) [35, 36], further support the notion that cAMP signaling restrict glial activation.

### cAMP signaling confers a in vivo-like transcriptional profile to cultured astrocytes

In contrast to other neural cell types, such as neurons and oligodendrocytes, astroglial cells grown in culture do not accurately reflect their attributes in vivo. For instance, cultured astrocytes display a high proliferative rate and deficient glutamate clearance, and they lack cell processes and membrane K^+^ and Cl^−^ conductances [7, 8]. It has been revealed that in vitro astrocytes show an immature, somewhat reactive, transcriptional profile [21, 37] (see also overlap between developing and in vitro sets in Fig. 4c). Therefore by comparing our results with gene sets of Cahoy et al. [21], we also examined whether cAMP-induced changes make the features of cultured astrocytes more like those observed in vivo. Surprisingly, 334 out of 1147 genes upregulated by cAMP were significantly enriched in the mRNA signature of brain-purified astrocytes (in vivo set), whereas a 32,5 % of cAMP-downregulated genes were common with the upregulated set of cultured astrocytes (in vitro set) (Fig. 4a-c). For instance, the connexin *Gja1*, the immunosuppressor *Nr4a2*, the antioxidant *Cat* and the Notch pathway effector *Hes5* were overexpressed by cAMP, whereas a strong downregulation was found for many annexins, caveolins and IGFBPs. The full gene lists are shown in Additional file 3: Table S7 and S8. Moreover, around two-thirds of the overlapped genes in the cAMP-upregulated/mature and cAMP-downregulated/developing groups were shared with in vivo (i.e. *Adra2a*, *Grin2c*, and *Kcnn2*) and *in vitro* (i.e. collagens, nidogens and kinesins) enriched gene sets, respectively. Our observation that the transcriptome of cultured astrocytes acquires a greater resemblance to that of in vivo cells when these cells are exposed to 8Br-cAMP may indicate that cAMP signaling confers astrocytes an in vivo-like phenotype by repressing activation and promoting differentiation. Because astrocytes cultured in serum-free conditions and in a bioactive3D system more closely resemble astrocytes in vivo [38, 39], an involvement of cAMP signaling in the environment-dependent acquisition of a in vivo phenotype of cultured astrocytes could be suggested.

### A transcriptional database of cAMP-regulated genes involved in astrocyte maturation

**Fig. 5:**
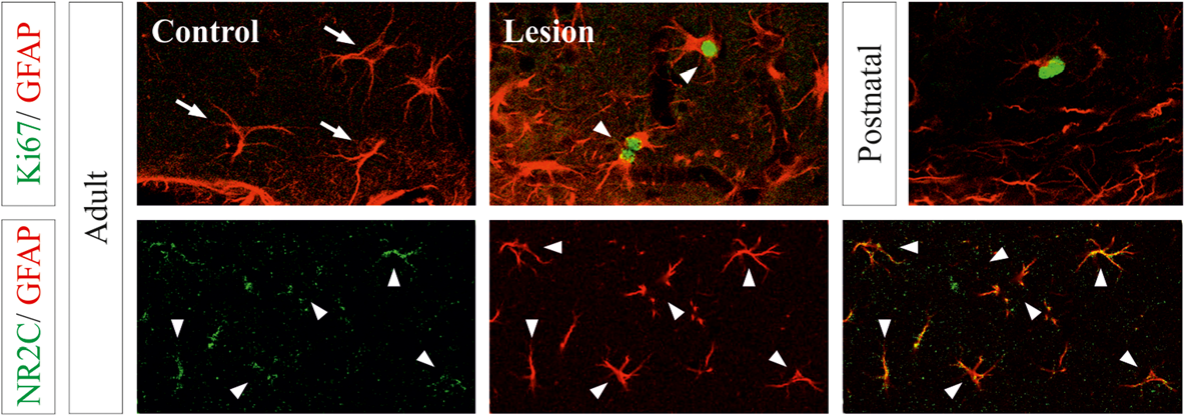
*In situ* expression of astroglial genes controlled by cAMP in the cerebral cortex in different states. Confocal images of double immunofluorescences of Mki67, Nestin, NR2C and GFAP in the hippocampal dentate gyrus (Mki67/GFAP) and the *stratum radiatum* of the CA1 region (NR2C/GFAP), and upper layers of neocortex (Mki67/Nestin) of postnatal and adult brains. Cortical lesions were analyzed 5 days after a stab wound injury in the contralateral (control) and ipsilateral (lesion) hemispheres. Arrows point to identified astrocytes expressing only GFAP. Arrowheads identify double labeled cells. Crosses and the asterisk indicate the lateral ventricle and the brain surface, respectively. Scale bar: 10 μm

We have contrasted our gene profiles with previous studies describing the transcriptome signatures of acutely isolated astrocytes [21, 28]. Next, we evaluate expression of representative genes regulated by cAMP *in situ* in cortical astrocytes in different states. *Mki67* and *Nes* genes were downregulated by cAMP in our cultures (−5 and −4.2 fold), but upregulated in acute-purified developing and reactive astrocytes [21, 28]. Conversely, mRNA expression of the NMDA receptor subunit NR2C was upregulated by cAMP (3.9 fold) as well as in isolated matured astrocytes [21]. In agreement with the proliferative state of cells, many nuclei of GFAP- and Nestin-positive astrocytes were immunolabeled for Mki67 in histological sections of postnatal and injured adult cortex, but not in the intact adult brain (Fig. 5). Although recent studies have shown that NR2C mRNA increases with age in cortical astrocytes *in situ* [40, 41], the location of NMDA receptor protein in glial cells in vivo is unknown. Here we show the protein expression of NR2C subunit in many astrocytes in the adult cerebral cortex (Fig. 5). In conclusion, the present GSEA results may provide a comprehensive database of transcripts involved in the acquisition of a mature astrocyte phenotype regulated by the cAMP pathway.

The induction of target genes and cellular programs regulated by the cAMP pathway is highly dependent on cellular contexts [42]. For instance, elevated cAMP results in cell-cycle arrest in astrocytes but induces proliferation of neurons [9, 43]. We postulate that the potent gene regulation by cAMP in astrocytes shown here is conveyed through PKA and the subsequent activation of the CREB transcription factors. Moreover, the recruitment of specific CREB regulatory partners, the activation of PKA-independent pathways and a secondary gene regulation may also collaborate to shape the individual astrocyte transcriptome elicited by cAMP signaling [43, 44].

## Conclusions

We here show that the net effect of cAMP signaling is to restrict developmental and activation features of astrocytes and promote their maturation. Therefore, we propose that controlled positive modulation of cAMP signaling could be used to repress the mechanisms of activation driven by pathological situations and to promote the physiologically normal state of differentiated astrocytes to support and protect neuronal populations.

## Methods

### Animals

OF1 mice were provided by Charles River Laboratories, Inc. (Lyon, France), kept under controlled temperature (22 ± 2 °C), humidity (40–60 %), and light (12-h cycles) and treated in accordance with the European Community Council Directive (86/609/EEC) on animal welfare. Under ketamine/xylazine anesthesia, stab wound lesions were made in the parietal cortex with a scalpel blade as described previously [19].

### Astrocyte cultures

Cultures were prepared from the cerebral cortex of 2-day-old mice [17]. Most cell culture reagents were obtained from GIBCO (Invitrogen, Paisley, UK). Briefly, the cerebral cortex was isolated and the meninges were carefully dissected out. Cortical tissues were then minced and incubated in 0.25 % trypsin and 0.01 % DNase. Dissociated cells were seeded in flasks and grown in high-glucose Dulbecco’s Modified Eagle’s Medium and F-12 (1:1) containing 10 % FBS, 10 mM HEPES and penicillin/streptomycin at 37 ° C in a 5 % CO_2_ incubator. At confluence (days 10–12), the flasks were shaken overnight and the cells were rinsed, detached and subcultured onto poly-L-lysine-coated plastic culture dishes and coverslips. Treatment with 1 mM 8Br-cAMP (Biolog, Life Science Institute, Bremen, Germany) was administered for 8 days to induce the robust morphological and physiological changes described elsewhere [7, 8].

### RNA Extraction and Reverse Transcription

RNA was isolated from triplicate cultures from the four individual experiments. Extractions were performed using using Trizol® Reagent (Invitrogen™) following the manufacturer’s instructions. The quantity and quality of isolated RNAs were determined with a NanoDrop ND-1000 (NanoDrop Technologies, Wilmington, DE, USA) and a Bioanalyzer 2100 (Agilent, Waldbronn, Germany). The RNA integrity numbers (RIN) in all cases ranged from 8.7 to 10, thereby indicating minimal RNA degradation [45]. First-strand DNA was synthesized using the Superscript III Reverse Transcriptase kit (Invitrogen). 1 μg of total RNA was added to kit components and nuclease-free water to 20 μl. Reactions containing nuclease-free water instead of enzyme served as a negative control. Reactions were incubated at 25 °C for 10 min, 50 °C for 30 min, 85 °C for 5 min. They were then chilled on ice. *E.coli* RNAse H was added and incubated at 37 °C for 20 min. The samples were then cooled to 4 °C and finally stored at −20 °C.

### Microarray Analysis

RNA (500 ng) was labeled using Agilent’s Low Input RNA Labeling Kit, which involves reverse transcribing the mRNA in the presence of T7-oligo-dT primer to produce cDNA and then in vitro transcribing with T7 RNA polymerase in the presence of Cy3-CTP or Cy5-CTP to produce labeled cRNA. The labeled cRNA was hybridized to the Agilent Mouse 4x44K 60-mer oligo microarray according to the manufacturer’s protocol. The arrays were washed, dried by centrifugation and scanned on an Agilent G2565BA microarray scanner at 100 % PMT and 5 μm resolution. Data were extracted using Genepix 6.0 (Molecular Devices) software using the irregular spot finding feature.

### Microarray Statistical Processing

Log2ratio values were computed for all pairs of control and cAMP-stimulated samples. Per-probe log2ratios were aggregated to per-gene values by taking median values. Analysis for differential expression on a gene-by-gene basis was performed by limma [46] while distinguishing biological from technical replicates (dye-swap hybridizations). Correction for multiple testing was done using the False Discovery Rate (FDR) method. Additional file 1: Table S1 with heat map was generated with Array File Maker 4.0 tool [47]. The lists of regulated genes were brought into biological context by Gene Ontology scoring using GSEA [48]. Also, for cross-study comparisons GSEA, was applied. This approach was used to test gene sets of interest (defined as lists of differentially expressed gene symbols derived from other studies) for significant enrichment among differentially expressed genes within our study. Only studies performed on mouse from which astrocyte cultures were obtained and maintained under similar conditions to those in the present study were considered. To compare the gene sets by Venn diagrams, we fixed the same fold change cut-off at 1.5.

### Quantitative Real-Time PCR Analysis

RT-qPCR was carried out using Power SYBR® Green PCR Master Mix (Applied Biosystems, Foster City, CA) on an Applied 7700 machine, using the primers indicated in Additional file 4: Table S9, and following the manufacturer’s protocol. A dilution series (10^0^–10^−3^) of total cDNA was prepared to determine the standard curve (relative quantification). The samples (in triplicates) were amplified according to the following protocol: 10 min at 95 °C, 42 cycles: 15 s at 95 °C, 15 s annealing temperature (60 °C), 30 s at 72 °C. To control the specificity of the reaction, melting-curve analysis was performed after amplification. In addition, PCR products were analyzed by agarose gel electrophoresis to confirm the size of the amplified targets. Levels of Tbp were used as normalization controls and relative mRNA levels were calculated as indicated by Pfaffl et al. [49] and expressed as mean ± standard error.

### Western blotting

Cultured cells were lysed in ice-cold 50 mM Tris–HCl, pH 7.4, 150 mM NaCl, 5 mM MgCl_2_, 1 mM EGTA, 1 % Triton-X 100 and protease inhibitor cocktail (Roche Diagnostics). Samples were electrophoresed in 10 % SDS-PAGE (BioRad) and then transferred to nitrocellulose membranes (Whatman® Schleicher & Schuell, Keene, NH). The membranes were blocked in a solution containing 5 % non-fat milk powder in TBST (140 mM NaCl, 10 mM Tris/HCl, pH 7.4 and 0.1 % Tween 20) for 1 h at room temperature and then incubated with primary antibodies (Additional file 5: Table S10) in blocking buffer for 2 h at room temperature. After several washes in blocking solution, the membranes were incubated for 1 h with HRP-conjugated secondary antibodies (DAKO, Glostrup, Denmark). Bound antibodies were visualized with enhanced chemiluminescence reagents PIERCE® ECL Western Blotting Substrate (Thermo Scientific, Rockford, IL, USA).

### Immunocytochemistry

Cells grown on glass coverslips were fixed with 4 % paraformaldehyde in PBS for 15 min. Adult and postnatal (P5) animals were perfused transcardially under deep anesthesia with the same fixative. Brains were removed from the skulls, postfixed for 4 h and cryoprotected overnight at 4 °C by immersion in a 30 % sucrose solution. Forty-micrometer-thick frozen sections were obtained with a cryostat and collected in PBS. To suppress nonspecific binding, cell cultures and brain sections were incubated in PBS containing 10 % FBS, 0.1 % Triton X-100, 0.2 % glycine, and 0.2 % gelatin for 1 h at room temperature. Incubations with the primary antibodies (Additional file 5: Table S10) were carried out overnight at 4 °C in PBS containing 1 % FBS, 0.1 % Triton X-100, and 0.2 % gelatin. Cell cultures and brain sections were processed for immunofluorescence using secondary fluorochrome-conjugated antibodies (Alexa Fluor 488 and Alexa Fluor 568, Molecular Probes, Eugene, OR). Cell nuclei were stained with 4′,6-diamidino-2-phenylindole (DAPI, Molecular Probes). Cell-containing coverslips and histological sections were mounted with Mowiol. Double immunofluorescences were performed by using primary antibodies raised in different species. Images were obtained with a Leica TCS SPE scanning confocal microscope. The specificity of the immunostaining was tested by omitting the primary antibodies or by replacing them with an equivalent concentration of nonspecific IgG. No immunostaining was observed in these conditions.

### Ethics statement

Animal experimental procedures were approved by the Ethics Committee of the Universitat de Barcelona, in compliance with Catalan and European legislation All efforts were made to minimize the number used and animal suffering.

## Additional files

Additional file 1: Table S1. Full list of significantly regulated genes in cAMP-treated cells. Differential expression analysis on a gene-by-gene basis was done using linear models (limma). The table contains all genes with FDR q-values <5 % and absolute fold changes > 1.2 and is sorted on the basis of fold changes in decreasing order. The first eight columns show a color representation of the log2ratios for each two-color hybridization of a cAMP-treated together with a control sample. The sign of dye-swap hybridization log2ratios (indicated by ‘DS’ in the column name) was flipped, such that values have the same “direction” as in the direct hybridizations, i.e. red (green) indicates up- (down-) regulation in cAMP-treated as compared to Control. The 9th column is the average log2ratio between cAMP-treated and Control samples. In addition to gene symbol and gene description, the remaining three columns show (symmetric) fold changes, limma p-values and FDR q-values. (XLS 4198 kb) Additional file 2: Figure S1. Barplot of enriched Gene Ontology categories. The barplot shows negative log10 q-values of Gene Ontology categories significantly enriched (q-value <0.02) with regulated genes according to GSEA analysis. The greater the bar height, the more significant the enrichment of the respective GO category. The vertical line indicates a q-value of 0.02. Enrichment analysis was done separately for downregulated genes (green bars) and upregulated genes (red bars). Categories are grouped into the three ontologies, as indicated within the bar labels (MF: Molecular Function, CC: Cellular Component, BP: Biological Process). The bar widths are proportional to the numbers of genes associated with the respective GO categories. The solid-colored part of the bars indicates the fraction of genes within the “core enrichment” of the respective category, as defined by GSEA analysis. (PDF 49 kb) Additional file 3: Tables S2–S8. Lists of genes regulated in cAMP-treated cells and present in published astrocyte signatures. Genes that are regulated in cAMP-treated cells (genes within the “core enrichment” as defined by GSEA analysis), and that are reported to be regulated in previously published astrocyte signatures: developing (Table S2), mature (Table S3), cytokine-treated (Table S4), LPS (Table S5), MCAO (Table S6), in vivo (Table S7) and in vitro (Table S8) astrocytes. The lists represent the respective intersections in the Venn diagrams of Figure 4C. In GSEA analysis, genes were ranked on the basis of signal-to-noise ratio within the microarray data, starting with strongly upregulated and ending with strongly downregulated genes. The third column of the tables indicate the rank of the respective gene, i.e. low rank values indicate strong upregulation and high ones close to the maximal value of 21,492 indicate strong downregulation. (XLS 108 kb) Additional file 4: Table S9. Primers used in this study. (XLSX 11 kb) Additional file 5: Table S10. Antibodies used in this study. (XLSX 11 kb)

## Abbreviations

FDR: false discovery rate
GFAP: glial fibrillary acidic protein
GSEA: gene set enrichment analysis
NES: normalized enrichment score
NR2C: NMDA receptor subunit 2C.
