# Supplementary figures and images for: Cyclic AMP signaling restricts activation and promotes maturation and antioxidant defenses in astrocytes
Source: BMC Genomics. 2016 Apr 23;17:304. doi: 10.1186/s12864-016-2623-4 (PMC4842285; doi:10.1186/s12864-016-2623-4)

Supplementary Figure 1

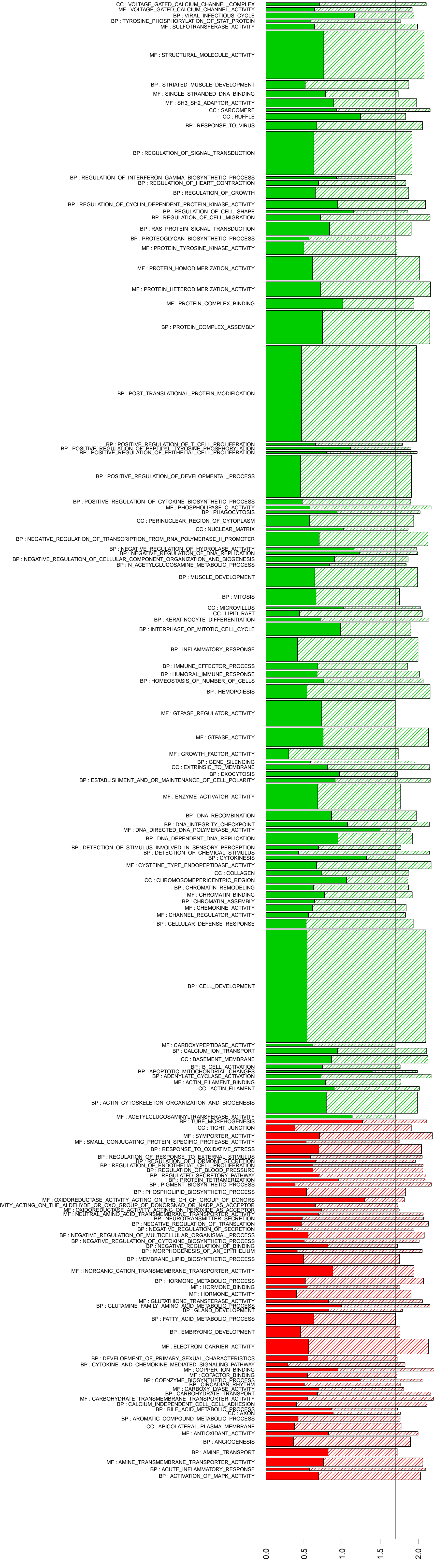

Supplement: Additional file 2: Figure S1. — Barplot of enriched Gene Ontology categories. The barplot shows negative log10 q-values of Gene Ontology categories significantly enriched (q-value <0.02) with regulated genes according to GSEA analysis. The greater the bar height, the more significant the enrichment of the respective GO category. The vertical line indicates a q-value of 0.02. Enrichment analysis was done separately for downregulated genes (green bars) and upregulated genes (red bars). Categories are grouped into the three ontologies, as indicated within the bar labels (MF: Molecular Function, CC: Cellular Component, BP: Biological Process). The bar widths are proportional to the numbers of genes associated with the respective GO categories. The solid-colored part of the bars indicates the fraction of genes within the “core enrichment” of the respective category, as defined by GSEA analysis. (PDF 49 kb) [file 12864_2016_2623_MOESM2_ESM.pdf]
